# Influence of antimicrobial prophylaxis in horses undergoing sutured castrations

**DOI:** 10.1111/vsu.14256

**Published:** 2025-03-31

**Authors:** Ida Sjöberg, Isabella Horn, Karl Ljungvall, Pia H. Andersen, Susanna Sternberg‐Lewerin

**Affiliations:** ^1^ Department of Clinical Sciences Swedish University of Agricultural Science (SLU) Uppsala Sweden; ^2^ Halland Animal Hospital Slöinge Sweden; ^3^ Department of Animal Biosciences SLU Uppsala Sweden

## Abstract

**Objective:**

To investigate the influence of surgical antimicrobial prophylaxis (SAP) on complication rates and surgical site infections (SSI) in horses undergoing sutured castration in a hospital setting.

**Study design:**

Retrospective chart review of a convenience sample.

**Animals:**

A total of 220 colts and stallions admitted for sutured castration.

**Methods:**

Medical records of sutured castration were assessed for patient characteristics, perioperative and postoperative medications, and postoperative complications within 6 weeks after surgery. The effect of these variables on the occurrence of complications was analyzed using χ^2^ tests, and the association between SAP use and SSI probability was evaluated.

**Results:**

The median age of horses included in the study was 3 years (range 1–14 years), and the overall complication rate was 10.0% (22/220). Surgical antimicrobial prophylaxis was administered to 62% of the patients (136/220). There were no differences in the overall complication rates, with a 7.1% (6/84) complication rate among horses without SAP *(SAP‐)* and an 11.8% (16/136) rate in those receiving SAP (*SAP+*) (*p* = .36). There was no association between the use of SAP and the probability of SSI, with an incidence of 3.6% in *SAP‐* and 4.4% in *SAP+* (3/84 and 6/136 respectively) (*p* = 1.0).

**Conclusion:**

Administering SAP did not influence the overall complication rate or the SSI rate following sutured castrations in a hospital setting.

**Clinical Significance:**

These findings prompt a reconsideration of the use of SAP for sutured castrations under aseptic conditions. The results may also serve as a basis for future randomized controlled trials.

**Abbreviations**
AMR: antimicrobial resistance
AN: anesthesia
NI: noninfectious
NSAID: nonsteroidal anti‐inflammatory drugs
SAP: surgical antimicrobial prophylaxis
SSI: surgical site infections
STB: Standardbred trotter
WBL: warmblood horse

## 1 INTRODUCTION

Surgical antimicrobial prophylaxis (SAP) has been used to prevent postoperative surgical site infection (SSI) since penicillin was developed in the 1940s. However, the emergence of antimicrobial resistance (AMR) is recognized as one of the most important threats to human and animal health worldwide, and indirectly to the global economy.^1^ Antimicrobial drug use, particularly overuse or misuse, drives resistance, leading to restrictions on SAP use.^2^ When SAP is used, the correct timing relative to the start of surgery, dosage, and choice of antimicrobial are all important for achieving the best prophylactic effect.^3^ However, antimicrobial drugs should never replace meticulous atraumatic and aseptic surgical techniques. Clean surgical procedures do not require SAP in general, unless the incidence of SSI exceeds 5% without SAP, or when there is a risk of devastating consequences if deep SSI occurs in the area.^3^, ^4^ Guidelines and policies for how and when to use SAP have been implemented in veterinary practices to encourage responsible use of antimicrobials. Although awareness of good antimicrobial stewardship is increasing, 48% of equine surgeons who responded to a British questionnaire still used SAP for clean surgeries.^5^ This was believed to be due to a lack of guidelines for many surgical procedures in horses. Benzylpenicillin is one of the most commonly used antibiotics for SAP.^6^ There is a limited choice of antimicrobial substances available for equine surgeons due to regulations and the risk of side effects such as acute colitis,^7^, ^8^ so preserving the efficacy of benzylpenicillin is particularly important.

Castration of stallions is one of the oldest and most common surgical procedures in horses. Open, closed, and semiclosed techniques have been described and castration can be performed with the patient standing or recumbent. Surgical incisions can be left open to drain and heal by secondary intention,^9^, ^10^, ^11^ or sutured for primary intention healing.^12^, ^13^, ^14^ Sutured castration under general anesthesia should be classified as a clean surgery according to the previously established wound‐classification criteria^6^ and, as such, it is not in need of SAP. The likelihood of SSI depends on several factors including the virulence of the endogenous skin flora, immune status and age of the horse, and the duration of surgery.^6^, ^15^ To reduce SSI in castrations, the combination of standard antiseptic surgical technique and closure of the incision is crucial.^6^ The definition of SSI is inconsistent in the available literature but the reported prevalence of SSI for sutured castrations appears to be in the region of 0.4% to 2.1%.^16^, ^17^, ^18^ However, SAP has been used in all of these reports and there is a lack of research focusing on complication rates and SSI without SAP.

The aim of this retrospective study was to investigate the influence of SAP on complication rates and SSI in sutured castrations of stallions performed under general anesthesia in a hospital setting.

## 2 MATERIALS AND METHODS

### 2.1 Medical records

A retrospective chart review of sutured castrations performed at an equine hospital, during 2016–2023 was conducted. One of the authors (IS) collected data from electronic medical records including age and breed of the horse, the month in which it was castrated, and perioperative and postoperative medication. Any notation of a postoperative complication in the medical record up to 6 weeks after castration was registered. All horses included in the study were castrated under general anesthesia by the same surgeon (IH), using a scrotal approach. Horses castrated with a different technique, by another surgeon, or horses with a urogenital pathology were excluded from the study. Horses were also excluded if prophylactic antimicrobials were not administered prior to surgery but within 24 h after surgery. All records were anonymized and due to the retrospective review of medical records only, institutional ethical review was not deemed necessary.

### 2.2 Anesthesia and surgical protocol

All horses received a single preoperative dose of a nonsteroidal anti‐inflammatory drug (NSAID), either meloxicam 0.6 mg/kg or flunixin 1.1 mg/kg IV. They were premedicated with acepromazine, an alpha‐2 agonist and butorphanol, followed by induction with benzodiazepine and ketamine, and anesthesia was maintained with isoflurane in oxygen. Horses were placed in dorsal recumbency, and the scrotal area was prepared for surgery by clipping if necessary, washing with chlorhexidine soap, and disinfecting with povidone‐iodine and ethanol. A sterile surgical drape covered the surgical area and the rear end of the horse. Local anesthetics were not used routinely.

All horses were castrated using a straight skin incision through the scrotal median raphe, followed by sharp and blunt dissection to the parietal layer of the vaginal tunic. The spermatic cord, including the cremaster muscle, was crushed with a noncutting, Sand‐type emasculator, followed by a double transfixed, encircling ligature with USP 2 polyglactin 910 in the crush zone. The spermatic cord was cut with a No 23 or 24 scalpel blade approximately 1 cm distal to the ligature to excise the testicles. The scrotal cavity was closed in two layers and the skin was closed with an intracutaneous suture to reduce dead space, all in a continuous pattern with USP 2–0 polyglactin 910. Surgery time was less than 60 min. The incision was protected with a water‐resistant wound spray (Opsite, Smith and Nephew, Mölndal, Sweden) and covered with a sterile gauze dressing. If no complication was observed after recovery, horses were discharged later on the same day or the day after. Recommendations for postoperative care were box rest with hand walking for 20 min twice a day for 3 days, followed by 1 week of turnout in a small paddock and extended exercise in walk for less than 60 min by hand, ridden, or in a cart and thereafter 1 week of light work in trot. After that, normal work could gradually be introduced. Owners were requested to check the rectal temperature daily and to report any complication such as fever, excessive swelling or lameness.

### 2.3 Definition of complications

Complications, summarized from the medical record, were categorized according to predefined definitions:

*Noninfectious (NI)*—excessive scrotal swelling without fever (i.e., hemorrhage, seroma or hematoma).

*Surgical site infection (SSI)*—excessive scrotal swelling with fever (>38.4°C), or purulent drainage from incision, or devitalized and infected tissue at repeat surgery, or positive culture on bacteriology.

*Anesthesia (AN)*—any catastrophic event during anesthesia or recovery; fever within 48 h after surgery without any clinical signs from the surgical site or within 2 weeks after surgery; signs of respiratory disease or colic.

### 2.4 Statistical analysis

Descriptive data related to age, breed, perioperative and postoperative medication, the month in which the castration was performed, and complication rates were analyzed. Univariable χ^2^ tests were performed to evaluate the effect of each of these variables on the occurrence of complication and SSI. The effect of antimicrobial prophylaxis was further analyzed and the castrations were grouped into horses that did not receive any antimicrobials *(SAP‐)* and horses that received perioperative antimicrobials *(SAP+)*. The significance level was set to *p* < .05. All analyses were conducted using the statistical Software JMP Pro16 (SAS Institute, Cary, North Carolina).

## 3 RESULTS

Horses (*n* = 220) with a median age of 3 years (range 1–14 years) were included. For statistical analysis, age was transformed into three age categories: 2 years or younger, 3–4 years, and 5 years or older (Table 1). The population consisted of 49% Standardbred trotters (STB) (*n* = 107), 30% warmblood horses (WBL) (*n* = 65), 8% ponies (*n* = 17) and 14% horses of miscellaneous other breeds (*n* = 31). Castrations were performed all year round. All horses followed the same anesthetic protocol and all received NSAIDs before surgery.

**TABLE 1:** Distribution of perioperative antimicrobial medication and complications in different age categories and breeds, *N* = 220.

Age and breed	Antimicrobial regimen			Complications			
	SAP‐ (%)	SAP+ (%)	*Total*	NI (%)	SSI (%)	AN (%)	Total (%)
Total	84 (38)	136 (62)	*220*	11 (5.0)	9 (4.1)	2 (0.9)	*22 (10.0)*
Age							
≤2	41 (39)	64 (60)	*105*	4 (3.8)	4 (3.8)	2 (1.9)	*10 (9.5)*
3–4	27 (33)	54 (67)	*81*	6 (7.4)	5 (6.2)	0 (0.0)	*11 (13.6)*
≥5	16 (47)	18 (53)	*34*	1 (2.9)	0 (0.0)	0 (0.0)	*1 (2.9)*
Breed							
STB	5 (5)	102 (95)*	*107*	7 (6.5)	6 (5.6)	1 (0.9)	*14 (13.1)*
WBL	45 (69)*	20 (31)	*65*	2 (3.1)	2 (3.1)	1 (1.5)	*5 (7.7)*
Pony	16 (94)*	1 (6)	*17*	0 (0.0)	0 (0.0)	0 (0.0)	*0 (0.0)*
Other	18 (58)	13 (42)	*31*	2 (6.5)	1 (3.2)	0 (0.0)	*3 (9.7)*

Abbreviations: AN, anesthetic category; NI, noninfectious category; SAP, surgical antimicrobial prophylaxis; SSI, surgical site infection category; STB, Standardbred trotter; WBL, warmblood horse.

* Denotes significant difference between SAP groups (*p* < .05).

Surgical antimicrobial prophylaxis with benzylpenicillin was administered to 62% of the castrated horses (*n* = 136) 60–120 min prior to the start of surgery. Of these, 103 horses continued to receive prophylactic antibiotics for a median duration of 3 days (range 2–6 days). Nonsteroidal anti‐inflammatory drugs were given postoperatively to 52% of all horses (*n* = 115) for a median duration of 5 days (range 3–6 days).

The overall complication rate was 10% (22/220) (Table 1). In a univariable analysis, age, breed, and time of year for surgery were not associated with complications, nor were perioperative or postoperative medications. In the NI category, 11 horses were included and median time from castration to first clinical signs of complication was 12 days (range 0–27 days). For the nine horses with complications categorized as SSIs, median time to first clinical signs was 17 days (range 6–33 days) after castration. Bacteriological cultures were obtained from eight cases, six in the SSI category and two in the NI category. There were two horses in the AN category, one with postoperative fever and one with colic. The Supporting Information, Table S1, presents a more detailed description of clinical signs and treatment of complications.

**FIGURE 1:**
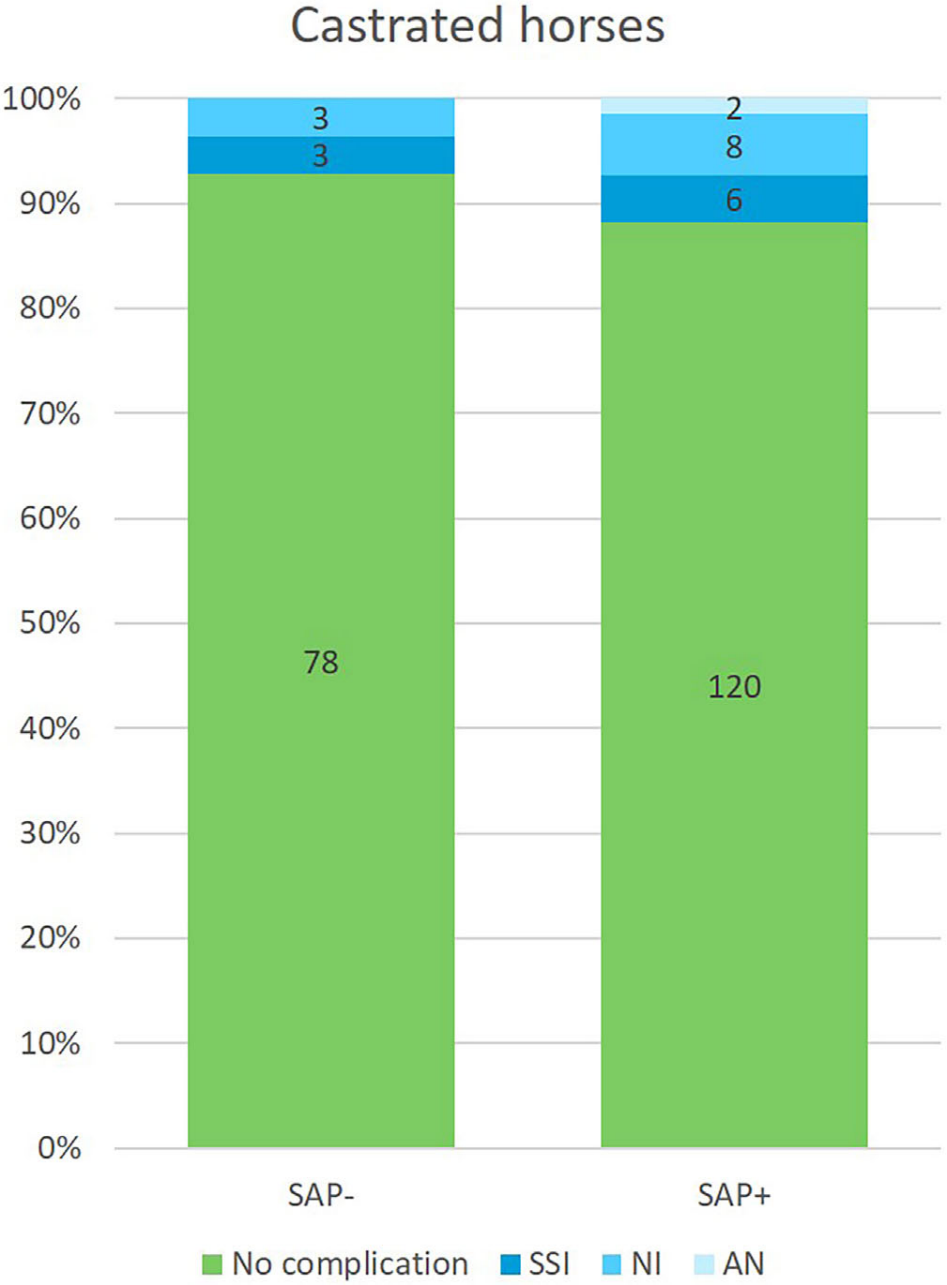
Castrated horses with and without surgical antimicrobial prophylaxis (SAP) and number of complications within the surgical site infection (SSI), noninfection (NI) and anesthesia‐related (AN) categories.

For further analysis of the effect of SAP, the castrations were grouped into *SAP‐* (*n* = 84) and *SAP+* (*n* = 136) (Table 1). There was a difference in the distribution of breeds between the two groups (*p* < .05), with more ponies and WBL in the *SAP‐* group and more STB in the *SAP+* group, but the age categories were similar. There was no association between groups and complication rates, with an incidence of 7.1% (6/84) in *SAP‐* and 11.8% (16/136) in *SAP+* (*p* = .36) (Figure 1). The use of SAP was not associated with SSI, with an incidence of 3.6% (3/84) in *SAP‐* compared with 4.4% (6/136) in *SAP+* (*p* = 1.0). Postoperatively, NSAIDs were prescribed to 93% of the horses in the *SAP‐* group (78/84) and to 27% in the *SAP+* group (37/136) (*p* < .05), but neither complication nor SSI rate in the total population was affected by postoperative use of NSAID (*p* = .51 and *p* = 1.0 respectively).

## 4 DISCUSSION

The results from this study indicate that SAP did not influence complication rates or SSI following sutured castrations performed under general anesthesia in a hospital setting. However, the surgeon's perception of the risk of SSI may have affected the use of SAP and thus also the reported complication rate.

Excessive swelling of the surgical site is the most common complication after castration, regardless of the technique.^17^, ^19^ Mild swelling is a reaction to the tissue trauma caused by the surgery but when the swelling becomes more severe and painful complications such as hemorrhage, seroma, or infection must be considered. Categorizing the swelling as septic or nonseptic is not straightforward, contributing to the discrepancies when complications are reported.^20^

In this study, complications categorized as NI did not present with fever but the swelling, in most cases, was treated by providing drainage and wounds were left open to heal by secondary intention. To prevent infection following open drainage, prophylactic use of antimicrobials was administered for variable durations. It is difficult to know for sure that these cases were not primarily infected and only two cases were sampled for bacterial culture.

In comparison, complications in the SSI category all presented with swelling of the surgical site, or fever, and/or had a positive bacterial culture. The risk of false negative culture results must be considered, especially if a horse is receiving antimicrobial treatment. In the present study, most cultures were positive, regardless of concurrent treatment with antimicrobials. The rate of SSI in this study was 4.1%, which is higher than reported in some other studies^16^, ^17^, ^18^ but still below the 5% threshold for using SAP. The results from this study did not support the assumption that SSI was associated with devastating consequences.

A majority (6/9) of horses diagnosed with SSI responded well to medical treatment combined with drainage of the surgical site. Only four horses needed to be reoperated under general anesthesia and eventually all horses were discharged from the hospital. This finding is in agreement with several other studies.^12^, ^16^ With a 3.6% incidence of SSI in the group of horses that did not receive SAP, this study indicates that the use of SAP is not beneficial in sutured castrations and not in accordance with the accepted guidelines for antibiotic use.^4^

In veterinary textbooks on castration techniques, the need for SAP is often considered to be questionable or up to the discretion of the surgeon.^11^, ^21^ This primarily applies to castration techniques where the wounds are left open for secondary intention healing, which inevitably leads to contamination of the surgical site. Sutured castrations are clean surgeries, generally completed within 60 min and performed in healthy, young animals. These are all low risk factors for SSI.^6^, ^22^ As previously stated, antimicrobials should not compensate for the lack of aseptic surgical technique.^3^, ^23^ Despite this, published research from the last three decades on sutured castration without SAP remains limited, with only two early descriptions of castrating stallions with primary closure,^13^, ^14^ and one recent study by Riemersma et al.^23^ The emerging threat of AMR poses a substantial risk to the ability to treat equine infections, especially in hospital settings. As any exposure to antimicrobials contributes to the development of AMR, it is crucial to use them only when required. The efficacy of commonly used substances, such as benzylpenicillin, must be preserved and they should not be used for unnecessary prophylaxis. Instead, the focus should be on hygiene measures and atraumatic techniques to prevent infections. Action plans set up in response to AMR largely aim to reduce antimicrobial use to support the “One Health” principle but also the wellbeing of the individual animal.^1^

The main limitation in this study lies in the retrospective study design. There was no standardized follow up, and complication rates may be underreported as some clients may have chosen to seek care from another veterinary facility if complications occurred. Medical records were extracted over a 7 year period, so it was deemed unlikely that owners/trainers would remember convalescence details. No attempt was made to contact them at the time of data collection. All castrations were performed by the second author, which means that another surgeon could have had a different outcome. On the other hand, a single surgeon provides consistency in technique and skills between the two groups.

There were differences between the two groups that need to be acknowledged. Standardbred trotters were overrepresented in the SAP group. Administration of SAP was determined at the surgeon's discretion, and there is a risk of selection bias that must be considered. Horses perceived to have a greater risk of SSI may have received antimicrobials to a greater extent than others. However, client expectations are a known factor contributing to the misuse of antimicrobials.^24^, ^25^ In the trotting industry there is a strong tradition among owners and trainers to give penicillin during castrations, which could have influenced the use of antimicrobials.

As evidence‐based guidelines for SAP in sutured castration are lacking, there is a risk of harming the relationship between client and surgeon if a postoperative complication occurs when SAP has not been used. There is a reluctance in the trotting industry to administer oral medication, especially NSAIDs, because of doping regulations and the risk of contaminating the environment. This could explain why NSAIDs were prescribed to almost all castrations in *SAP‐*, but only to one fourth of horses in the *SAP+* group. However, neither breed nor NSAID use was associated with the occurrence of complications when looking at the whole study population.

Another limitation is that the author collecting the data was aware of the objectives of the study. This could potentially bias data collection and estimation of SSI. To avoid bias, the definitions of the three complication categories were predefined and the operating surgeon was not involved in the process. Conflicting data were discussed with the other members in the group until consensus was reached.

Ideally, a larger sample size would have allowed for a more robust statistical analysis. The total number of horses was deemed sufficient for noninferiority testing, with an expected rate of SSI of 1.1% and an accepted maximum difference of 3.9%. Nevertheless, there is a possibility that the groups were too small to detect minor differences. However, this study contributes to the limited existing knowledge of the prevalence of complications and SSIs following sutured castration without SAP, and may serve as a foundation for future randomized controlled trials.

In conclusion, the use of SAP was not associated with complication rates or the probability of SSI in sutured castrations in this study population. The prevalence of SSI was less than 5% regardless the use of SAP and most complications resolved without a second surgery. The results from this study can guide equine surgeons towards a decision not to use SAP routinely in sutured castrations and thereby reduce the amount of antimicrobials used in equine surgeries. Further studies into the decision making of equine surgeons regarding the use of SAP would be useful.

## FUNDING INFORMATION

This study is a part of a doctoral program financed by a grant from the Swedish University of Agricultural Science. Preliminary results of this study were presented as a poster at the European College of Veterinary Surgeons (ECVS) Annual Scientific Meeting, Valencia, Spain, July 4–6, 2024.

## CONFLICT OF INTEREST

All authors declare that they have no known competing financial interests or personal relationships that could have influenced the work reported in this paper.

## Supporting information


**Table S1.** Description of cases with a complication including categorization, pre‐ and postoperative medications, history, clinical signs and treatment.
