# Supplementary material for: Influence of antimicrobial prophylaxis in horses undergoing sutured castrations
Source: Vet Surg. 2025 Mar 31;54(5):872–8. doi: 10.1111/vsu.14256 (PMC12282032; doi:10.1111/vsu.14256)
Supplement: Supplementary file 1 — Table S1. Description of cases with a complication including categorization, pre‐ and postoperative medications, history, clinical signs and treatment. [file VSU-54-872-s001.docx]

**Table S1.** Description of cases with a complication including categorization, pre- and postoperative medications, history, clinical signs and treatment.

| **Case** | **Breed** | **Age** | **Ab pre op** | **Ab post op (d)** | **NSAID post op (d)** | **Compl** | **History and clinical signs** | **Treatment** | **Bacterial culture** |
| --- | --- | --- | --- | --- | --- | --- | --- | --- | --- |
| 6 | WBL | 2 | Benzyl pc | None | Meloxicam (6) | SSI | d16; fluctuating scrotal swelling  d20; drainage from surgical wound, no fever | d16; TMS  d20; drainage of surgical wounds and flushed with saline, Benzyl pc and flunixin for 7 days, thereafter TMS for another 5 days | Positive for Streptococcus |
| 8 | STB | 3 | Benzyl pc | None | Meloxicam (5) | SSI | d17; scrotal swelling, fever 39.0℃  d21 still scrotal swelling, fever and hind limb lameness | d17; pc, increased exercise  d21; drainage of surgical wounds. Pc, gentamicin and flunixin for 7 days, continued with pc and meloxicam another 6 days | Positive for Streptococcus |
| 9 | STB | 4 | None | None | None | SSI | Slight scrotal swelling since castration.  d27; moderate swelling and fever, 39.0℃ | d27 pc 10 days and meloxicam 5 days | Not performed |
| 50 | OTH | 3 | None | None | Meloxicam (6) | SSI | Moderate scrotal swelling 7 days after castration, no fever. Treated with 2 courses of Pc.  d39; severe swelling | d39; drainage of surgical wounds  d40; resection of devitalized tissue and drainage of deep seroma formation under general anesthesia | Positive for Staphylococcus |
| 52 | STB | 2 | Benzyl pc | None | Meloxicam (5) | SSI | d6; scrotal swelling and fever 40.0℃.  d29; fistula formation with purulent discharge from drainage wounds | d6; drainage of surgical wounds, treated with flunixin and firstly pc and secondly with oxytetracycline  d29; resection of devitalized tissue and funiculi under general anesthesia | Positive for Streptococcus |
| 60 | STB | 2 | Benzyl pc | Procaine pc (3) | None | SSI | d33; moderate scrotal swelling with slight purulent discharge, no fever | d33; treatment with TMS for 5 days | Not performed |
| 175 | WBL | 4 | None | None | Meloxicam (4) | SSI | d17; severe scrotal swelling, fever 40.0℃ | d20; drainage of surgical wounds and flushed with saline repeatedly, pc, gentamicin and flunixin for 10 days, continued with pc another 7 days | Positive for Streptococcus |
| 180 | STB | 1 | Benzyl pc | Procaine pc (5) | None | SSI | d6; slight scrotal swelling and fever. | d6; treatment with pc for another 5 days | Not performed |
| 214 | STB | 3 | Procaine pc | Procaine pc (3) | None | SSI | d20; moderate hard scrotal swelling with fever >40.0℃ | d20; pc and meloxicam, not responding  d21; suspicion of hematoma, treated with pc, gentamicin and meloxicam, not responding  d26; resection of devitalized tissue under general anesthesia, continued treatment with pc for 6 days | Negative |
| 5 | STB | 2 | Benzyl pc | Procaine pc (5) | None | NI | d27; moderate scrotal swelling, no fever | Increased exercise | Not performed |
| 7 | STB | 4 | Benzyl pc | Procaine pc (2) | None | NI | Scrotal swelling after castration. Treated with Procaine pc by local vet.  d30; severe swelling | d30; drainage of surgical wounds, large amount of serohemorrhagic fluid. Treatment with Procaine pc | Negative |
| 31 | STB | 4 | Benzyl pc | Procaine pc (5) | None | NI | Slight scrotal swelling since castration.  d10; severe, hard scrotal swelling, no fever | d10; drainage of surgical wounds, pc 5 days, meloxicam 7 days | Not performed |
| 32 | STB | 3 | Benzyl pc | Procaine pc (3) | None | NI | d0; moderate scrotal swelling after recovery  d2; increased swelling, suspected hemorrhage | d2; drainage of surgical wounds, blood clots excavated, the cavity was flushed with saline, pc, flunixin and exercise | Not performed |
| 51 | STB | 4 | Benzyl pc | Procaine pc (3) | None | NI | d5; moderate scrotal swelling, no fever | d5; drainage of surgical wounds, pc | Negative |
| 53 | STB | 3 | Benzyl pc | None | None | NI | d22; slight scrotal swelling and some clear discharge, no fever.  d29; severe swelling, no fever. | d22; TMS 3 days,  d29; drainage of surgical wounds, large amount of hemorrhagic fluid, TMS 14 days and NSAID 4 days | Not performed |
| 54 | STB | 3 | Benzyl pc | Procaine pc (3) | None | NI | d7 moderate scrotal swelling | d7; increased exercise | Not performed |
| 130 | OTH | 1 | None | None | Meloxicam (5) | NI | d15; seroma formation  d26; hard scrotal swelling, with no fever | d15; drainage of seroma in scrotum, meloxicam  d26; pc and meloxicam 7 days | Not performed |
| 211 | WBL | 2 | None | None | Meloxicam (5) | NI | d16; moderate scrotal swelling without fever. | d16; increased exercise | Not performed |
| 213 | WBL | 1 | None | None | Meloxicam (5) | NI | d12; slight scrotal swelling and some wound discharge, without fever | d12; increased exercise | Not performed |
| 246 | OTH | 7 | Benzylpc | Procaine pc (3) | Meloxicam (5) | NI | d1; severe scrotal swelling, without fever.  d13; continuous serohemorrhagic discharge from wounds. | d2; suspected hemorrhage, drainage of surgical wounds and flushed with saline, continue pc for 5 days  d13; repeated drainage and wounds flushed with saline, pc, gentamicin and local treatment with pc, but not responding  d20; Resection of devitalized tissue and funiculi under general anesthesia. Continued treatment with TMS for 5 days | Not performed |
| 190 | STB | 2 | Benzylpc | Procain pc (5) | No | AN | d0; mild clinical signs from the respiratory tract. d1; fever >39.0℃, no clinical signs from the castration site. | No additional treatment | Not applicable |
| 248 | WBL | 2 | Benzylpc | None | Meloxicam (5) | AN | d0; mild colic. | d0; mild impaction at rectal examination. Responded well to treatment with metamizole and fluids over night. | Not applicable |

Ab; antibiotics, AN; anesthetic related, Compl; complication category, d; days, NI; non-infectious, NSAID; non-steroidal anti-inflammatory drug, op; operative, OTH; Other breed, pc; penicillin, SSI; surgical site infection, STB; Standardbred trotter, TMS; trimethoprim sulfadiazine, WBL; warmblood horse
